# Brazilian version of the Functional Status Score for the ICU: translation and cross-cultural adaptation

**DOI:** 10.5935/0103-507X.20170006

**Published:** 2017

**Authors:** Vinicius Zacarias Maldaner da Silva, Jose Aires de Araújo Neto, Gerson Cipriano Jr., Mariela Pinedo, Dale M. Needham, Jennifer M. Zanni, Fernando Silva Guimarães

**Affiliations:** 1 Programa de Ciências da Saúde, Escola Superior de Ciências da Saúde - Brasília (DF), Brasil.; 2 Hospital de Base do Distrito Federal - Brasília (DF), Brasil.; 3 Hospital Santa Luzia Rede D'Or São Luiz - Brasília (DF), Brasil.; 4 Programa de Pós-graduação em Ciências da Reabilitação, Centro Universitário Augusto Motta - Rio de Janeiro (RJ), Brasil.; 5 Departamento de Fisioterapia, Universidade Federal do Rio de Janeiro - Rio de Janeiro (RJ), Brasil.; 6 Divisão de Pneumologia e Terapia Intensiva, Johns Hopkins University School of Medicine - Baltimore, MD, Estados Unidos.; 7 Grupo "Outcomes After Critical Illness and Surgery" (OACIS), Johns Hopkins University - Baltimore, MD, Estados Unidos.; 8 Programa de Doutorado em Ciências da Saúde e Tecnologia, Departamento de Fisioterapia, Universidade de Brasília - Brasília (DF), Brasil.

**Keywords:** Translations, Validation studies, Surveys and questionnaires, Intensive care units

## Abstract

**Objective:**

The aim of the present study was to translate and cross-culturally adapt the Functional Status Score for the intensive care unit (FSS-ICU) into Brazilian Portuguese.

**Methods:**

This study consisted of the following steps: translation (performed by two independent translators), synthesis of the initial translation, back-translation (by two independent translators who were unaware of the original FSS-ICU), and testing to evaluate the target audience's understanding. An Expert Committee supervised all steps and was responsible for the modifications made throughout the process and the final translated version.

**Results:**

The testing phase included two experienced physiotherapists who assessed a total of 30 critical care patients (mean FSS-ICU score = 25 ± 6). As the physiotherapists did not report any uncertainties or problems with interpretation affecting their performance, no additional adjustments were made to the Brazilian Portuguese version after the testing phase. Good interobserver reliability between the two assessors was obtained for each of the 5 FSS-ICU tasks and for the total FSS-ICU score (intraclass correlation coefficients ranged from 0.88 to 0.91).

**Conclusion:**

The adapted version of the FSS-ICU in Brazilian Portuguese was easy to understand and apply in an intensive care unit environment.

## Abstract (pt)

**Objetivo:**

Traduzir e adaptar culturalmente a Escala de Estado Funcional em UTI (FSS-ICU - *Functional Status Score for the ICU*) para o português do Brasil.

**Métodos:**

O presente estudo consistiu das seguintes fases: tradução (realizada por dois tradutores independentes), síntese da tradução inicial, tradução de volta ao inglês (realizada por dois tradutores independentes não familiarizados com a FSS-ICU original) e fase de teste, para avaliar a compreensão por parte da audiência alvo. Um comitê de especialistas supervisionou todas as fases e foi responsável pelos ajustes ao longo do processo e pela versão final da tradução.

**Resultados:**

A fase de testes incluiu dois fisioterapeutas experientes que avaliaram um total de 30 pacientes críticos (escore da FSS-ICU médio de 25 ± 6). Como os fisioterapeutas não relataram problemas com incertezas ou problemas de interpretação que afetassem seu desempenho, não foram feitos outros ajustes à versão em português brasileiro após a fase de teste. Obteve-se uma boa confiabilidade entre observadores para cada uma das cinco tarefas da FSS-ICU e entre os escores dos dois avaliadores (o coeficiente de correlação intraclasse variou de 0,88 a 0,91).

**Conclusão:**

A versão adaptada da FSS-ICU para o português brasileiro comprovou ser de fácil compreensão e aplicação clínica no ambiente da unidade de terapia intensiva.

## INTRODUCTION

Critically ill patients commonly develop muscle weakness and functional impairments that may persist long after discharge from the intensive care unit (ICU).^(1,2)^ Consequently, there is a need for objective measurement tools that assess a patient's ability to complete basic mobility tasks. This type of functional data may be beneficial to determining rehabilitation strategies in this setting.^(3)^

The use of standardized outcome measures in physical therapy has been accepted as best practice.^(4)^ In recent years, a number of assessment tools have been developed to assist in the evaluation of physical function in critically ill patients.^(5)^ Zanni et al.^(6)^ published the Functional Status Score for the ICU (FSS-ICU) and used this scale to describe the functional impairments of patients receiving treatment in intensive care. The FSS-ICU measures mobility tasks, including rolling, transferring from supine to sitting, transferring from sitting to standing, sitting on the edge of the bed and walking. The total FSS-ICU score ranges from 0 to 35, with higher scores indicating more independent physical functioning.

A recent systematic review has shown that most assessment tools employed by health professionals to evaluate functional outcomes in the ICU were originally developed in English.^(3)^ Therefore, research in countries where the official language is not English, such as Brazil, is hampered with regard to the use of these tools, especially when only a literal translation has been employed.^(7)^ For this reason, cross-cultural adaptation is an ideal choice for assessment tools available in the medical field, as this process allows for the tool to be applied in any country, culture and language. In addition, cross-cultural adaptation facilitates the comparison of results from the same questionnaire in different countries and cultures.^(8)^ Thus, the aim of the present study was to translate and cross-culturally adapt the FSS-ICU into Brazilian Portuguese.

## METHODS

### Study design

The translation and cross-cultural adaptation of the FSS-ICU into Brazilian Portuguese followed the current guidelines recommended for this type of study.^(8)^ The authorization for this process was obtained from the senior author of the original version, Dr. Dale M. Needham at Johns Hopkins University, Baltimore, USA. This study received approval from the Ethics Committee of *Fundação de Ensino e Pesquisa em Ciências da Saúde* (FEPECS - Brasilia-Brazil) under process number 1.338.188.

### Description of the Functional Status Score for the ICU

The FSS-ICU is an outcome measure assessing physical function that is specifically designed for patients in the ICU and involves five functional tasks (rolling, supine to sit transfer, sit to stand transfer, sitting on the edge of bed and walking). Each task is evaluated using an 8-point ordinal scale ranging from 0 (not able to perform at all) to 7 (complete independence) (Table 1; the instrument, scoring details and the full Brazilian Portuguese version are freely available at www.ImproveLTO.com). This instrument has been internationally validated with a detailed psychometric evaluation^(9,10)^ and has been used in other studies in the field.^(10-13)^ Scores on the FSS-ICU scale at ICU discharge have been shown to predict post-ICU hospital length of stay and hospital discharge location.^(9,10)^

**Table 1:** Example Scale for Scoring the Functional Status Score for the ICU*

Score	Definition
0	Unable to attempt or complete task due to weakness
1	Complete dependence
2	Maximal assistance (patient performing ≤ 25% of work)
3	Moderate assistance (patient performing 26% - 74% of work)
4	Minimal assistance (patient performing ≥ 75% of work)
5	Supervision only
6	Modified independence
7	Complete independence

* Full details of the Functional Status Score for the ICU administration and scoring, including a description of the scoring scales and the 5 functional tasks (rolling, transferring from supine to sitting, sitting on the edge of the bed, transferring from sitting to standing, and walking) are freely available at www.ImproveLTO.com.

### Translation and cross-cultural adaptation

The questionnaire and instructions were translated into Portuguese by two bilingual (Portuguese and English) translators whose native language was Brazilian Portuguese. One of the translators (T1) had experience in health terminology and was familiar with occupational issues regarding task assessments. The other translator (T2) had no experience in health care or knowledge of occupational task assessments. Both translators produced independent translations (T1 and T2).

### Translation synthesis (T1 + T2)

The independently translated versions of the FSS-ICU were compared and analyzed. A consensus approach was used to resolve any differences via meetings between the translators and coordinator. This process resulted in a consensus-based translation of the questionnaire (T1 - 2).

### Back-translation to original language

The synthesized Brazilian-Portuguese version was then back-translated into English by two other independent translators fluent in Portuguese and English (BT1 and BT2). The translators were not familiar with the concepts explored in the questionnaire and had no knowledge of the original English version. All five versions (T1, T2, T1-2, BT1 and BT2) were revised by the Translation Board, which included the author of the original FSS-ICU, three physical therapists, a bachelor in Arts & Linguistics, and all four translators. The Translation Board discussed each item, searching for the best solution to address the discrepancies and the different translation options. Rather than focusing on indices of agreement, the Translation Board attempted to capitalize on the language expertise of its members, resolving the following types of disagreements: conceptual (referring to the conceptualization of the assessment), idiomatic (different linguistic expressions), semantic (differences related to the test content), and experiential (cultural differences). After this process, the Translation Board produced a pre-final version of the FSS-ICU. This version was tested by two qualified physical therapists who had received standardized training in the FSS-ICU. The assessments were performed independently, and the physical therapists were blinded to the other's score. Patients were then screened for eligibility and asked to participate. The objective of this phase was to identify interpretation problems regarding the operational, conceptual, semantic and idiomatic equivalences of the items with the aim of enhancing the inventory as well as reviewing and modifying problematic questions. The participants constituted a convenience sample of inpatients in the cardiovascular and trauma ICUs. All patients provided written informed consent. Participants were included if they were admitted to an ICU, were more than 18 years old, had been mechanically ventilated for more than 48 hours and were expected to remain in the unit for at least four days. As the patients had to cooperate with the assessment, scores of at least 3 out of 5 using the De Jonghe comprehension criteria (*open and close your eyes; look at me; open your mouth and stick out your tongue; nod your head; raise your eyebrows when I have counted up to five*)^(14)^ on two consecutive occasions within a six-hour period were required as an inclusion criterion. Patients were excluded if they had physical or cognitive impairment that would prevent exercise or were admitted with a new neurological condition, such as stroke or spinal cord injury. Assessments were performed upon discharge from the ICU.

### Data analysis

A 1-sample Kolmogorov-Smirnov test was used to test the normality of the data. The mean ± standard deviation of the variables was calculated.

Intraclass correlation coefficients (ICCs) were calculated to evaluate the reliability between the two evaluators. An ICC greater than 0.75 was considered to indicate good to excellent reliability.^(15)^

## RESULTS

Thirty patients enrolled in the present study. Table 2 displays their demographic characteristics.

**Table 2:** Characteristics of the population submitted to the pretest - Functional Status Score for the ICU - Brazilian Version (n = 30)

Variables	
Age (years)	56 (14)
Male	15 (50)
APACHE II, mean (SD)	16 (8)
ICU admission diagnosis	
Respiratory (including pneumonia)	15 (50)
Gastrointestinal	2 (6)
Sepsis, non-pulmonary	3 (10)
Cardiovascular	5 (16)
Trauma	2 (6)
Neurological	3 (10)
FSS-ICU score	25 (6)

ICU - intensive care unit; APACHE II - Acute Physiology and Chronic Health Evaluation II. Values are expressed as the mean (standard deviation), number (%) or mean ± standard deviation.

During the preparation of the T1 and T2 versions, a high degree of semantic agreement was found between the translators. For Item 1.2, which asks "*Does the patient require cueing or coaxing in order to roll?"*, the word "coaxing" was translated as *incentivo* or *estímulo* in these two versions; however, the word *estímulo* did not remain after the board review.

In the back-translation, differences were found when compared to the original version. For Item 4, the phrase 'Supine to Sit Transfers' in the original was back-translated as 'Transfers from supine to a seated position'. All other items are summarized in table 3. In the pretest step, the physiotherapists did not report that any uncertainties or problems with interpretation affected their performance; therefore, no additional adjustments were made to the Brazilian Portuguese version. The interobserver reliability results are shown in table 4. There was very good interobserver reliability between the two assessors for all tasks and for the total FSS-ICU score.

**Table 3:** Translation by translator 1 and translator 2 and final version of the Functional Status Score for the ICU - Brazilian version

Item	Original version	T1	T2	T12
1	*If a task was not performed due to patient weakness…*	Se a tarefa não foi realizada devido à fraqueza do paciente	Se a(s) tarefa(s) não foi realizada por outra razão que não seja a fraqueza	Se a(s) tarefa(s) não foi realizada por outra razão que não seja a fraqueza
1.3	*Does the patient require cueing or coaxing in order to roll…*	O paciente precisa de incentivo verbal ou orientação	O paciente precisa de estímulo verbal ou orientação	O paciente precisa de incentivo verbal ou orientação
2	*Does the patient require assistance to come to sitting from supine position?*	O paciente necessita de assistência para se sentar de uma posição deitado para sentado?	O paciente precisa de assistência para se sentar partindo de uma posição deitada?	O paciente precisa de assistência para se sentar partindo de uma posição deitada?
2.3	*Does the patient require cueing or coaxing in order to be able to come to sitting from a lying down position*	O paciente precisa de incentivo verbal ou orientação para ir de uma posição deitada para uma posição sentada, apesar de ser fisicamente capaz	O paciente precisa de incentivo verbal ou orientação para ir de uma posição deitada para uma posição sentada, apesar de fisicamente ser capaz	O paciente precisa de incentivo verbal ou orientação para ir de uma posição deitada para uma posição sentada, apesar de ser fisicamente capaz
2.5	*Does the patient require minimal assistance to come to sitting from a lying down position (defined as the patient performing 75% or more of the amount of the work?)*	O paciente precisa de assistência máxima para ir de uma posição deitada para uma posição sentada (definida como o paciente que realiza 75% ou mais do trabalho total)?	O paciente precisa de assistência máxima para ir de uma posição deitada para uma posição sentada (definida como o paciente que realiza 75% ou mais do esforço total)?	O paciente precisa de assistência máxima para ir de uma posição deitada para uma posição sentada (definida como o paciente que realiza 75% ou mais do trabalho total)?
5.2	*Does the patient require only supervision or coaxing to walk 150 feet (45m) without physical help?*	O paciente precisa somente de supervisão ou incentivo verbal para andar 150 pés (45m) sem ajuda física (o paciente pode usar um equipamento de assistência, se necessário)?	O paciente precisa somente de supervisão ou incentivo verbal para andar 150 pés (45m) sem ajuda (o paciente pode usar um equipamento de assistência, se necessário)?	O paciente precisa somente de supervisão ou incentivo verbal para andar 150 pés (45m) sem ajuda física (o paciente pode usar um equipamento de assistência, se necessário)?

T1 - translator 1; T2 - translator 2.

**Table 4:** Intra-class correlation coefficient values of the functional tasks of the Functional Status Score for the ICU

FSS-ICU task	Intra-class correlation coefficient (95%CI)
Rolling	0.84 (0.54 - 0.94)
Supine to sit transfer	0.86 (0.68 - 0.94)
Sit to stand transfer	0.85 (0.57 - 0.94)
Sitting on the edge of bed	0.90 (0.77 - 0.96)
Walking	0.91 (0.80 - 0.94)
FSS-ICU total score	0.88 (0.73 - 0.95)

FSS-ICU - Functional Status Score for the ICU; 95%CI - 95% confidence interval.

## DISCUSSION

This is the first study to complete an official translation and cross-cultural adaptation of the FSS-ICU into the Brazilian Portuguese language. The cross-cultural adaptation of specific questionnaires is not a simple task, as both language-related and cultural differences between countries should be considered to preserve the validity and reliability of assessment tools.^(16)^

The FSS-ICU has been used to evaluate physical function in the ICU environment. Mehrholz et al.^(12)^ found that the FSS-ICU score may predict the recovery of walking ability in people with ICU-acquired weakness. Thrush et al.^(11)^ evaluated functional status using the FSS-ICU within 4 days of ICU admission and every two weeks until discharge. They found that the FSS-ICU score significantly improved during ICU stays and that this tool may document functional improvements in ICU patients.

According to Parry et al.^(5)^ and the recent international validation of this instrument,^(9)^ which included data from Brazil, the USA and Australia, the FSS-ICU is recommended for evaluating patients in the ICU. Thus, the Brazilian Portuguese version of the FSS-ICU provides Brazilian physiotherapists an important assessment tool for clinical practice and research, considering its psychometric strengths, applicability and external validity. Huang et al.^(9)^ evaluated the internal consistency, validity, responsiveness and minimal important difference of the FSS-ICU scale from five international datasets, including two from Brazil. Moreover, the intrarater reliability has been assessed, identifying an intraclass correlation coefficient ≥ 0.985.^(10)^

The participation of different translators and back-translators in the early stages of the cross-cultural adaptation of the FSS-ICU was an important strategy to reduce the possibility of biases regarding the domains and items studied. Aspects of the scale that did not match with Brazilian culture underwent all relevant considerations raised by the professionals who performed the translations and back-translations.

The FSS-ICU is a tool that can be utilized to evaluate physical function in the ICU setting, requires no additional equipment, and can be easily integrated into clinical care by physiotherapists.

## CONCLUSION

The adapted version of the Functional Status Score for the Intensive Care Unit in Brazilian Portuguese proved to be easy to understand and apply clinically; however, it requires training and experience to be used in decision-making processes in the assessment of functional activities among patients in the intensive care unit.
